# Transdermal Diagnosis of Malaria Using Vapor Nanobubbles

**DOI:** 10.3201/eid2202.151829

**Published:** 2016-02

**Authors:** Ekaterina Lukianova-Hleb, Sarah Bezek, Reka Szigeti, Alexander Khodarev, Thomas Kelley, Andrew Hurrell, Michail Berba, Nirbhay Kumar, Umberto D’Alessandro, Dmitri Lapotko

**Affiliations:** Rice University, Houston, Texas, USA (E. Lukianova-Hleb, D. Lapotko);; Baylor College of Medicine, Houston (S. Bezek, R. Szigeti);; Ben Taub General Hospital, Harris Health System, Houston (S. Bezek, R. Szigeti);; X Instruments LLC, Fremont, California, USA (A. Khodarev);; Precision Acoustics Ltd, Dorset, England, UK (T. Kelley, A. Hurrell);; Standa UAB, Vilnius, Lithuania (M. Berba);; Tulane University, New Orleans, Louisiana, USA (N. Kumar);; Medical Research Council, Banjul, The Gambia (U. D’Alessandro);; London School of Hygiene and Tropical Medicine, London, UK (U. D’Alessandro)

**Keywords:** malaria, bloodless, hemozoin, haemozoin, nanobubble, laser, noninvasive, parasites, vector-borne infections, diagnosis, *Anopheles* spp., *Plasmodium falciparum*, malaria pigment

**In Response:** The letter by Rebelo et al. (*1*) that questions our previously described noninvasive malaria diagnostics (*2*,*3*) misinterprets both articles. The main objection comes to our alleged call for “large-scale studies in humans”; no such statement appeared in our 2014 article (*2*), and in the 2015 article (*3*), we clearly stated that large-scale studies will be considered after the optimization of a new prototype and improving its sensitivity. The authors’ final questioning of our eligibility for resources is a nonscientific opinion.

Concerning the quality of the standard clinical diagnosis, both thin blood film analysis and rapid diagnostic test results were obtained in a certified US clinical laboratory and returned consistent data. The lack of re-evaluation of the patient and the diagnostic timing are indeed limitations but were caused by the clinical restrictions. Our goal in the 2015 article (*3*) was to demonstrate the first noninvasive diagnosis of malaria in a human, which was achieved. The additional parameters discussed in the letter were not the subject of this study. Their letter further misinterprets our 2014 study, stating that parasitemia was virtual in that article; in fact, we studied actual infections among mice (*2*).

The criticism of Rebelo et al. might have been fueled by their own limited detection of hemozoin with flow cytometry and microscopy (*4*), in which they used parasite cultures and an unspecified number of malaria patients. That the methods they used might not have performed well does not mean that the novel technology we described, based upon a different mechanism, would have the same limitations in detecting hemozoin.

In conclusion, we agree with the need for optimization of the technology and additional testing. We are currently developing and testing our technology in a malaria-endemic country. Nevertheless, the letter by Rebello et al. does not alter the fact that our novel noninvasive malaria diagnostic technology worked in a human.

## References

[1] Rebelo M, Grenho R, Orban A, Hänscheid T. Transdermal diagnosis of malaria using vapor nanobubbles. [letter]. Emerg Infect Dis. 2016;22:343. 10.3201/eid2202.151203 PMC4734514 26811926

[2] Lukianova-Hleb EY, Campbell KM, Constantinou PE, Braam J, Olson JS, Ware RE, Hemozoin-generated vapor nanobubbles for transdermal reagent- and needle-free detection of malaria. Proc Natl Acad Sci U S A. 2014;111:900–5. 10.1073/pnas.1316253111 24379385 PMC3903264

[3] Lukianova-Hleb E, Bezek S, Szigeti R, Khodarev A, Kelley T, Hurrell A, Transdermal diagnosis of malaria using vapor nanobubbles. Emerg Infect Dis. 2015;21:1122–7. 10.3201/eid2107.150089 26079141 PMC4480396

[4] Rebelo M, Shapiro HM, Amaral T, Melo-Cristino J, Hänscheid T. Haemozoin detection in infected erythrocytes for *Plasmodium falciparum* malaria diagnosis-prospects and limitations. Acta Trop. 2012;123:58–61 . 10.1016/j.actatropica.2012.03.005 22465900

